# Disentangling early and late onset of psychosis in women

**DOI:** 10.1192/j.eurpsy.2022.904

**Published:** 2022-09-01

**Authors:** A. Díaz-Pons, A. González-Rodríguez, V. Ortiz-García De La Foz, M. Seeman, C. Facorro, R. Ayesa-Arriola

**Affiliations:** 1 Valdecilla Biomedical Research Institute, Psychiatry, Santander, Spain; 2 Mutua Terrassa University Hospital, Department Of Mental Health, Terrassa, Spain; 3 University of Toronto, Department Of Psychiatry, Toronto, Canada; 4 Hospital virgen del Rocio, Sevilla, Sevilla, Spain

**Keywords:** First Episode Psychosis, women, Early onset psychosis, Late onset psychosis

## Abstract

**Introduction:**

Women present a second peak of incidence of psychosis during menopausal transition, partially explained by the loss of estrogen protection conferred during the reproductive years. Despite this, few studies compare sociodemographic, biological, clinical varibles and neurocognitive performance between women with early onset of psychosis (EOP) and those with late onset of psychosis (LOP).

**Objectives:**

Our aim was to characterize both groups in a large sample of women, of which 294 were FEP patients (EOP = 205; LOP = 85) and 202 were healthy controls (HC) grouped following cutoff point (<>40 years of age) in previous studies.

**Methods:**

Clinical and laboratory assessments were completed. Neurocognitive performance was also evaluated, and a cognitive global deficit score (GDS) was derived. ANCOVA was used for comparisons.

**Results:**

EOP women were more frequently single and unemployed than comparable HC. Cholesterol levels in LOP women were higher than those of EOP women. LOP presented less severe symptoms, and higher scores in processing speed and premorbid IQ than EOP patients. Cannabis and alcohol use were also more frequent in EOP than LOP women.

**Figure:**
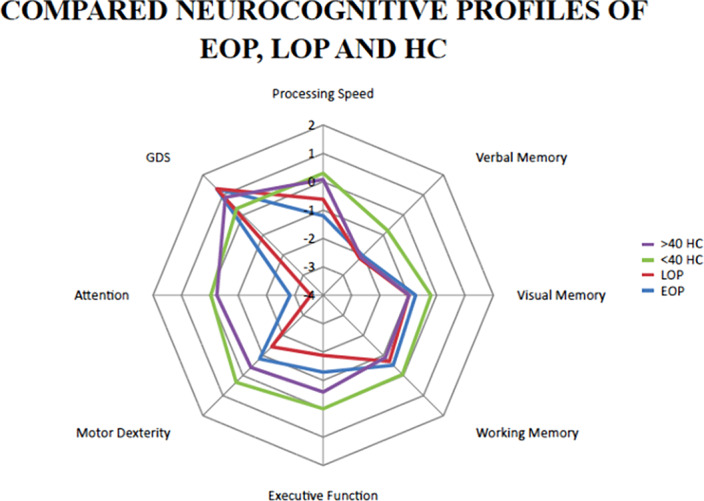

**Conclusions:**

Women with EOP and LOP show several sociodemographic, neuropsychological and clinical differences which may be valuable for planning personalized treatment emphasizing in socialization and differential generational dynamics. Some of these differences may be due to the aging process, while others might be influenced by factors such as lack of estrogen neuroprotection. In turn, drug consumption, low IQ and recent experienced trauma could as well reduce efficacy of hormonal neuroprotection.

**Disclosure:**

No significant relationships.

